# Supplementary material for: The Adult ADHD Self-Report Scale (ASRS): utility in college students with attention-deficit/hyperactivity disorder
Source: PeerJ. 2014 Mar 25;2:e324. doi: 10.7717/peerj.324 (PMC3970798; doi:10.7717/peerj.324)
Supplement: Supplemental Information 1 [file peerj-02-324-s001.docx]

| **Study** | **Objectives** | **Sample** | **Methods** | **Results** | **Limitations** |
| --- | --- | --- | --- | --- | --- |
| Kessler, R. C., et al. (2005).  The World Health  Organization Adult ADHD  Self-Report Scale (ASRS): a  short screening scale for use  in the general population.  *Psychological medicine*,  *35*(2), 245–56. | To evaluate a new adult self report measure for WMH surveys. | *N* = 154  *Age* = 18-44  *Sex* = nationally representative  *Sample* = Community:  US national comorbidity survey replication + oversample of those who reported ADHD symptoms as a child and continuing to adulthood. | In person interviews  1) ASRS v1.1  Diagnostic efficiency statistics:  Sensitivity, Specificity:  Total classification accuracy, Odds ratio, Cohen’s K, AUC  2) ASRS six-item screener: developed using step-wise logistic regression  3) Clinical interview: ADHD Rating Scale, Assessment of childhood ADHD, clinical interview DSM-IV, Self-report battery | 1) ‘Each ASRS symptom measure was significantly related to the comparable clinical symptom rating, but varied substantially in concordance (Cohen's K in the range 0.16-0.81).’  2) The unweighted six-question ASRS screener was superior to the unweighted 18-question ASRS in sensitivity, specificity, total classification accuracy, and k (0.76 v.0.58).  3) Six item ASRS: one-third of clinical cases would be missed with this screener, two-thirds in the highest stratum would be classified as having a very high probability of being cases.  4) 18 item ASRS: ‘refines prediction of the clinical classification among those who are positive on the six-question screener, correlates significantly with clinician-rated overall symptom severity.’ | -No informant data  -No identification of stimulant medication taken in sample  - Self reports filled out after clinician interview (possible sensitization to symptoms) |
| Kessler, R. C., et al. (2007).  Validity of the World Health  Organization Adult ADHD  Self-Report Scale (ASRS)  Screener in a representative  sample of health plan  members. *International*  *Journal of Methods in*  *Psychiatric Research*, *16*(2),  52–65. | Cross-validation of the ASRS Screener | *N* = 668  *Age* = 18+  *Sex* = nationally representative  *Sample* =  Community:  US managed care plan subscribers | T1: ASRS screener administered by phone  T2: convenience sample = 496 screened positive + 172 screened negative  T3: 155 screened positives + 63 screened negative given semi-structured clinical interviews. | 1) 6-item screener: ‘Principal axis  factor analysis found only the first  factor in each sample to have an  eigenvalue greater than 1.0 (1.4  2.0), with Cronbach’s α for the  factor-based scales in the range  .63–.72’  2) ‘Pearson correlations for  stability of scale scores over time  are consistently somewhat lower  for the 6 item screener than for the  0–24 scoring approach.’  3) ‘SEM analysis suggests that almost all of the inter-temporal instability in the ASRS Screener is due to measurement unreliability rather than to change in the true score.’ | -No reporting of medication (however excluded a subset who were ‘in treatment’ for ADHD)  - Clinical assessment: excluded ‘in remission’ adults not reaching 6/9 symptoms.  -No informant reports |
| Adler et al. (2006). Validity of Pilot Adult ADHD Self-Report Scale (ASRS) to Rate Adult ADHD Symptoms. *Annals of Clinical Psychiatry, 18(3),* 145-148. | To validate pilot ASRS against clinician ratings on the ADHD rating scale (ADHD RS).  *not able to access to full article | *N* = 60  *Age* = Adult | Cronbach's alpha  Agreement of raters: established by intra-class correlation coefficients (ICCs) between scales. | 1) ‘High internal consistency for both patient and rater-administered versions (Cronbach's alpha 0.88, 0.89)’  2) ICC between scales: .84  3) ‘ICCs for subset symptom scores were also high (both 0.83), acceptable agreement for individual items (% agreement: 43%-72%) and significant kappa coefficients for all items (p < 0.001)’ | *not able to access to full article |
| Yeh, C., Gau, S. S., Kessler, R.  C., & Wu, Y. (2008).  Psychometric properties of the  Chinese version of the adult  ADHD Self-report Scale.  *International Journal of*  *Methods in Psychiatric*  *Research*, *17*(1), 45–54. | To establish the normative data, reliability, and validity of the Chinese versions of the ASRS. | Sample 1:  *N* = 1031  *Age* = 22  *Sex* = Male  *Sample =* Chinese Army Base participants  Sample 2:  *N =* 3298  *Age* = 18  *Sex* = 62% male  first year College students | Measures: ASRS  WURS  Impulsiveness Scale  ICC for 2 subscales (IN/HY) on ASRS, and Cronbach’s Alpha.  Concurrent Validity: correlation with WURS | 1) ASRS separated into inattention/hyperactivity: Good concordance (intraclass correlations = 0.80 ∼ 0.85) and internal consistency (Cronbach's alpha = college sample: 0.83∼0.89 and army sample: 0.85∼0.91)  2) ‘Moderate to high correlations between these subscales and the WURS (Pearson's correlations = 0.37 ∼ 0.66).’ | - Male only sample for validity analysis  - No informant report  - Diagnosis based on subject report, no clinical assessment |
| Hines, J. L., King, T. S., &  Curry, W. J. (2012). The adult  ADHD self-report scale for  screening for adult attention  deficit-hyperactivity disorder  (ADHD). *Journal of the*  *American Board of Family*  *Medicine,* *25*(6),  847–53. | To analyze the ASRS for evaluating patients in a primary care setting. | *N* = 217 (ASRS), 55 (ASRS + CAARS)  *Age* = 18-65  *Sex* = 65% female  *Sample* = general primary care clinic, no ADHD diagnosis | Measures: 6-item ASRS  CAARS-S:S  Measured: sensitivity, specificity using contingency table analysis. | 1) Participation rate = 92%  2) Average time to complete = 54.3 seconds  3) Prevalence: 6%  4) High Sensitivity: 1.0  Moderately high specificity: 0.71 | - Prevalence based on positive ASRS + CAARS  - No examination of comorbidity  - No informant ratings |
| Zohar, A. H., & Konfortes, H.  (2010). *The Israel journal of*  *psychiatry and related*  *sciences*, *47*, 308–315. | To examine the properties of the ASRS v1.1 in Hebrew and test validity in college students. | *N* = 192  *Sex* = not reported  *Age* = 24  *Sample* = Israeli college volunteers and LD center students. 43 had ADHD diagnosis | ASRS v1.1 Hebrew  Computer version (items only appear after last item was answered) + paper version | 1) High test-retest reliability (.60-.90)  2) Cronbergs Alpha: ‘All reliability estimates are between 0.79 and 0.89’  3) Full scale outperforms the 6-item scale for sensitivity  4) Paper mode slightly better in reliability  5) ‘Participants with ADHD rated themselves higher on the ASRS_C than on the ASRS_P’ | - Test-retest was done only between modes, not over time  - Limited generalization from sample of college students |
| Hesse, M. (2013). The ASRS  6 has two latent factors:  attention deficit and  hyperactivity. *Journal of*  *attention disorders*, *17*(3),  203–7. | ‘To test two different factor structures for the ASRS-6’ | *N* = 234 (students), 157 (outpatient)  *Sex* = 40% male  *Age* = 25  *Sample* 1= Danish college students (BA, MA)  Sample 2 = outpatients treated for drug dependence | Measures: ASRS 6  DIP-Q  BSSS-4  Kessler-6  Confirmatory factor analysis | 1) ‘Across both samples, the two-factor model produced acceptable goodness-of-fit statistics, whereas the one-factor model failed to fit the data.’  2) The two factors are correlated and test re test, in the college sample is adequate. | - Limited generalization from sample of college students and outpatients treated for drug dependence  - No standard with which to compare to determine discriminant validity |
| Van de Glind, et al. (2013).  Validity of the Adult ADHD Self  Report Scale (ASRS) as a  screener for adult ADHD in  treatment seeking substance use  disorder patients. *Drug and*  *alcohol dependence*, *132*(3), 587  96. | To test the utility and performance of the ASRS for adult ADHD in a sample seeking treatment for substance use disorders. | *N = t1: 3558, t2 = 1138*  *Age =* 18-65  Sex = t2 26.0% female  Sample = seeking treatment for substance use disorder | ASRS 6 item  CAADID for external criterion (sensitivity, specificity, LR+, LR-, PPV, NPV) | 1) The overall positive predictive value was 0.26, negative predictive value was 0.97  2) The sensitivity was good and specificity moderate for indentifying possible ADHD cases in this population.  3) The ASRS was not a good screener for externalizing disorders other than ADHD. | - Large drop out rate between t1, t2  - No informant ratings |
| Ramos-Quiroga et al., 2009.  Validation of the Spanish version  of the attention deficit  hyperactivity disorder adult  screening scale (ASRS v. 1.1): a  novel scoring strategy. *Revista de*  *neurologia*, *48*(9), 449–52. | To examine a Spanish version of the 6-item ASRS  *not able to access to full article | *N =* 90 Control,  90 ADHD  Sample = Outpatient treatment program | Clinical diagnosis:  Connors Adult ADHD diagnostic Interview | 1) Using a cut off of 12 points (0-24 point system, scaling from 0-4), they found high sensitivity, specificity, Kappa index of .88, Area under the curve = 0.94 | *not able to access to full article |
